# Arboviruses isolated from the Barkedji mosquito-based surveillance system, 2012-2013

**DOI:** 10.1186/s12879-018-3538-2

**Published:** 2018-12-12

**Authors:** El Hadji Ndiaye, Diawo Diallo, Gamou Fall, Yamar Ba, Ousmane Faye, Ibrahima Dia, Mawlouth Diallo

**Affiliations:** 1 Unité d’Entomologie Médicale, Institut Pasteur de Dakar, 36 Avenue Pasteur, BP 220 Dakar, Senegal; 2 Unité des Arbovirus et Virus de Fièvres Hémorragiques, Institut Pasteur de Dakar, 36 Avenue Pasteur, BP 220 Dakar, Senegal

**Keywords:** Arbovirus, Surveillance, Mosquitoes, Aampling methods, Ponds, Villages

## Abstract

**Background:**

A mosquito-based arbovirus surveillance system was set up at Barkedji, Senegal after the first outbreak of Rift valley fever in West Africa in 1988. This system was recently updated using more sampling methods and collecting in greater number of ponds and villages sites.

**Methods:**

For the current study, mosquitoes were sampled biweekly between July and December 2012 and 2013 using CDC+CO_2_ light traps set at ground and canopy level, mosquito nets baited with goat, sheep, human or chicken, light traps baited with goat, sheep and chicken; bird-baited traps using pigeons or chickens placed either at the ground or canopy level. Collected mosquitoes were identified, pooled and screened for arboviruses.

**Results:**

A total of 42,969 mosquitoes in 4,429 pools were processed for virus isolation. Ten virus species were identified among 103 virus isolates. West Nile virus (WNV; 31 isolates), Barkedji virus (BARV; 18), Sindbis virus (SINV; 13), Usutu virus (USUV; 12), Acado virus (ACAV; 8), Ndumu virus (NDUV; 9), Sanar virus (SANV; 7), Bagaza virus (BAGV; 3), Rift valley fever virus (RVFV; 1), and Yaounde virus (YAOV; 1) were isolated from 9 ponds (91 strains) and 7 villages (12 strains). Only 3 virus species (WNV, NDU and SINV) were isolated from villages. The largest numbers of isolates were collected in October (29.1% of total isolates) and November (50.5%). Viruses were isolated from 14 mosquito species including *Cx. neavei* (69.9% of the strains), *Cx. antennatus* (9.7%), and *Ma. uniformis* (4.8%). NDUV, ACAV, and SINV are herein reported for the first time in the Barkedji area. Isolation of ACAV and SANV from a pool of male *Ma. uniformis* and USUV and BARV from a pool of male *Cx. neavei*, are reported for the first time to our knowledge.

**Conclusion:**

Our data indicate that the Barkedji area is characterized by a high diversity of viruses of medical, veterinary and unknown importance. Arboviruses were first detected in July at the beginning of the rainy season and peaked in abundance in October and November. The Barkedji area, an enzootic focus of several potentially emerging arboviruses, should be surveilled annually to be prepared to deal with future disease emergence events.

## Background

The Barkedji area, located in the Sahelian biogeographic area of Senegal is known as a focus of enzootic transmission of several arthropod-borne viruses (arboviruses). Indeed, a surveillance program set up in this area after the first outbreak of Rift valley fever in Senegal in 1988 [1, 2] resulted in the isolation of several viruses including 2 alphaviruses (Semliki Forest and Babanki), 6 flaviviruses (Barkedji (BARKV), Bagaza (BAGV), Usutu (USUV), Yaounde (YAOV), West Nile (WNV), Koutango and Saboya), 2 bunyaviruses (Bunyamwera, and Ngari), 2 phleboviruses (Rift valley fever (RVFV), and Gabek Forest), 1 orbivirus (Sanar (SANV)), 1 rhabdovirus (Chandipura) and 1 unclassified virus (ArD95537). Some of these arboviruses (RVFV, USUV, and WNV) are of medical and/or veterinary importance, while the potential health impact of the others is still unknown [2–7]. Enzootic cycles of these viruses generally involve mosquito vectors and domestic and/or wild vertebrates as amplifying or reservoirs hosts [5, 8]. Human are known to be incidentally affected by several of these viruses but are considered as dead-end hosts that do not support ongoing transmission. The viruses listed above cause clinical syndromes of varying severity, ranging from acute benign fevers of short duration to life-threatening encephalitis and/or hemorrhagic fever [8].

Arboviruses surveillance program can monitor viruses in vertebrate hosts and/or arthropod vectors. The surveillance of virus in vertebrate hosts has several drawbacks relative to surveillance of arthropods, including the limited accessibility of hosts, small sample sizes of hosts, and variation of susceptibility of different vertebrate species to the same virus [9]. Surveillance of mosquitoes for circulating arboviruses can be used as an early warning system because it allows the detection of seasonal initiation or increase of virus circulation. Thus, the implementation of mosquito surveillance can give decision makers enough time to enact efficient measures for outbreak control [10]. Mosquito-based virus surveillance programs are also important tools in the study of the eco-epidemiology and transmission of viruses by enabling identification of potential vectors of a given virus as well as the spatio-temporal dynamics of the virus. Mosquito surveillance can also be useful for identifying new arbovirus species of medical and veterinary importance [9, 11, 12].

The mosquito surveillance program for detection of arboviruses that has been active in Barkedji since 1990 used to collect mosquitoes each month using human landing catch, CDC+CO_2_ light trap set at the ground level, and animal + light baited traps in a very few temporary ponds and villages [1, 13]. This program resulted in the identification of all the arbovirus species listed above, the vectors of RVFV and description of the RVFV enzootic cycle. Previous studies, in the Barkedji area, have shown a high diversity of the mosquito fauna in the study area with a dominance of *Aedes vexans* and *Culex poicilipes* and a switch over of dominance of these two species between years [1, 14, 15]. They also showed that *Ae. vexans* was always the most abundant species at the beginning of the rainy season, while *Cx. poicilipes* dominated the end of the rainy season. These species preferred host seeking in barren and temporary ponds rather than villages and other land cover Classes were rare [15, 16]. These vectors are temporary ponds breeders [1, 14, 16, 17]. The maximum flight distances, from these ponds, of the main vectors were estimated, to be around 650 m for *Ae. vexans* and 550 m for *Cx. poicilipes* [16]. Diallo and others [15], predicted that *Ae. vexans*, *Cx. poicilipes* and *Cx. neavei* mosquitoes would not disperse up to 1,500 m to the nearest ponds. The seasonal dynamics of these vectors were positively correlated with rainfall for *Ae. vexans*, after a lag time of one month for the *Culex* species. All the vectors had their highest abundances and parity rates between September and November [15].

However, identification and description of arboviruses circulating in the area are still incomplete. To date, data are still lacking on spatial extent of arbovirus circulation and the complete suite of potential vectors for each virus. In 2012 and 2013, the program was modified to expand the number of trap types and baits used, increase the frequency of trapping from monthly to biweekly, and increase the number of ponds and villages sampled. This paper reports the result of this modified surveillance program in 2012 and 2013. Our specific objectives were to detect arboviruses circulating in the Barkedji area, their associated vectors and their spatio-temporal dynamics.

## Methods

### Study Area

**Fig. 1:**
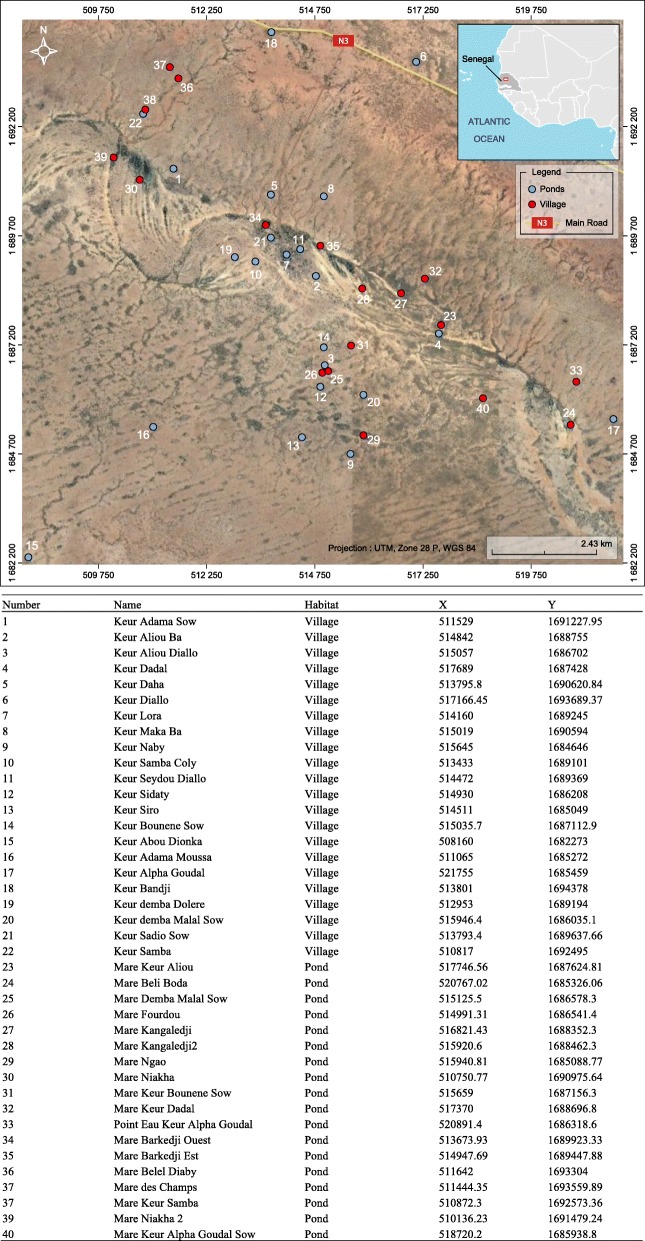
Study area with the names of villages and ponds sampled and their geographic coordinates in UTM (Modified from Google Earth)

The study was conducted in the Ferlo region of Senegal in an area located around the Barkedji village (14° 53' 0" N, 15° 55' 0" W) from late July to late December in 2012 and 2013. This area (Fig. 1) is semi-desert, characterized mainly as shrubby savanna, with a hot dry climate with a long and severe dry season and short rainy season that generally occurs between July and September. Annual rainfall is approximately 300 mm. A network of temporary ponds of different sizes is flooded by the first rains. Small ponds are flooded and drained after each rain. Large ponds are flooded at the beginning of the rainy season, remain inundated for a long period and are covered by hydrophytes. These ponds are the main source of water for herders and their livestock in this period. These ponds are also the natural habitats of many vertebrate species (birds, reptiles and rodents) and mosquito vectors of arboviruses. Horses and donkeys serve for transportation and animal traction. They congregate around ponds in the afternoon and at night. The area is covered with a rich carpet grass used by local and nomadic populations during the rainy season. The main activities of people are millet cultivation and sheep, goats, and cattle grazing. Family groups and their herds may become seasonal nomads, temporarily relocating for a few months during which they use grass huts for shelter. Barkedji is the only substantial and populated village in the area, while the other villages are located near ponds and composed of just a few huts.

### Mosquito collection

In 2012, mosquitoes were collected using animal+light-baited traps [18], mosquito net-baited traps [17] and CDC+CO_2_ light traps [19] set on the shore of 18 ponds, and using CDC+CO_2_ light traps in 22 villages around Barkedji. Mosquito were collected monthly in villages and biweekly in ponds. In 2013, ponds were sampled biweekly with the traps listed above as well as bird-baited traps [20]. In November 2013, 3 ponds and 1 village were sampled once in Thiargny (50 km from Barkedji) following suspicion of an arbovirus outbreak. The sampling methods used were CDC+CO_2_ light traps set at ground and canopy level; mosquito-net baited with goat, sheep, human or chicken; light trap baited with goat, sheep and chicken; bird baited trap using pigeons or chickens at the ground and canopy level. Each fortnight, traps were set from dusk to dawn. In the field laboratory, mosquitoes were sorted, identified and pooled (maximum of 89 and average of 9.8 mosquitoes per pool) by species, sex and collection site on a chill table using several morphological keys [21–26]. Mosquito pools were then stored in liquid nitrogen and shipped to the Institut Pasteur in Dakar for virus testing.

### Virus isolation and identification

Mosquito pools were homogenized in 3 ml of L-15 medium (Gibco BRL, GrandIsland, NY, USA), using chilled tissue-grinders, supplemented with 20% fetal bovine serum (Gibco) and clarified by centrifugation at 1500 g, 4°C for 10 min. Following centrifugation, the supernatant was filtered using a 1 ml syringe (Artsana, Como, Italy) and sterilized with 0.20 μm filters (Sartorius, Göttingen, Germany).

Following procedures described previously in Digoutte et al. [27], the clarified and filtered suspensions were inoculated into *Aedes pseudoscutellaris* (AP61) and Vero cell lines and incubuated for 7-8 days. The presence of virus was detected by indirect immunofluorescence using using in-house hyper-immune mouse ascites fluids directed to individual or groups of more than 70 African arboviruses (flaviviruses, bunyaviruses, orbiviruses and alphaviruses). Identifications were later confirmed by complement fixation and seroneutralization tests. All virological tests were carried out by the WHO Collaborating Center of Reference and Research on Arboviruses and Hemorrhagic Fever Viruses of the Institut Pasteur de Dakar.

### Data analysis

Mosquito seasonal distribution patterns were evaluated by measuring the absolute abundance of mosquitoes, quantified as the average females per trap (CDC+CO_2_ light traps only) per night. The proportion (%) of each mosquito vector collected in all traps per habitat was also calculated. The Minimum Field Infection Rate (MFIR ‰) was calculated as the number of positive pools per 1000 mosquitoes. Differences of frequencies between groups were tested by contingency table analyses. All analyses were conducted using R [28].

## Results

### Mosquito collection

A total of 42,992 mosquitoes (41,921 females) representing 7 genera and 42 species were collected (Table 1), including 1 species of *Aedomyia*, 15 *Aedes*, 7 *Anopheles*, 13 *Culex*, 2 *Mansonia*, 3 *Mimomyia* and 1 *Uranotaenia*. The predominant species in both the ponds (22.9% of all mosquitoes collected) and villages (49.1%) was *Ae. vexans*. This species was followed by *Ae. ochraceus* (11.2%) and *Cx. poicilipes* (9.0%) in villages and by *Cx. poicilipes* (19.7%), *Ma. uniformis* (11.0%) and *Cx. neavei* (10.9%) in ponds.

**Table 1:** Mosquito females collected in ponds and 694 villages in the Barkedji area, 2012-2013

Species	Ponds		Villages		Total	
	No collected (males)	%	No collected (males)	%	No collected (males)	%
*Aedomya africana*	7	0	0	0	7	0
*Aedes aegypti*	110 (20)	0.3	10	0.2	120 (20)	0.3
*Aedes argenteopunctatus*	258	0.7	14	0.3	272	0.6
*Aedes dalzieli*	1297 (3)	3.5	94	1.7	1391 (3)	3.2
*Aedes fowleri*	120	0.3	73	1.3	193	0.4
*Aedes furcifer*	205	0.5	1	0	206	0.5
*Aedes hirsutus*	29	0.1	1	0	30	0.1
*Aedes luteocephalus*	22	0.1	3	0.1	25	0.1
*Aedes mcintoshi*	5	0	3	0.1	8	0
*Aedes metallicus*	18 (1)	0	2	0	37 (1)	0.1
*Aedes minutus*	156	0.4	54	1	210	0.5
*Aedes ochraceus*	1377 (1)	3.7	627	11.2	2004 (1)	4.7
*Aedes sudanensis*	659 (9)	1.8	115	2.1	774 (9)	1.8
*Aedes unilineatus*	21 (1)	0.1	0	0	21 (1)	0
*Aedes vexans*	8546 (12)	22.9	2751	49.1	11297 (12)	26.3
*Aedes vittatus*	0	0	1	0	1	0
*Anopheles coustani*	1	0	0	0	1	0
*Anopheles funestus*	1	0	0	0	1	0
*Anopheles gambiae*	122 (8)	0.3	86	1.5	208 (8)	0.5
*Anopheles pharoensis*	256 (13)	0.7	37	0.7	293 (13)	0.7
*Anopheles rufipes*	15	0	96	1.7	111	0.3
*Anopheles squamosus*	54 (5)	0.1	44	0.8	98 (5)	0.2
*Anopheles ziemanni*	2396 (4)	6.4	250	4.5	2646 (4)	6.2
*Culex annulioris*	0	0	17	0.3	17	0
*Culex antennatus*	1264 (10)	3.4	114	2	1378 (10)	3.2
*Culex bitaeniorhynchus*	533 (1)	1.4	76	1.4	609 (1)	1.4
*Culex decens*	18	0	0	0	18	0
*Culex ethiopicus*	1107 (12)	3	55	1	1162 (12)	2.7
*Culex neavei*	4077 (459)	10.9	146	2.6	4223 (459)	9.8
*Culex nebulosus*	2	0	10	0.2	12	0
*Culex perfuscus*	525	1.4	22	0.4	547	1.3
*Culex poicilipes*	7382 (113)	19.7	502	9	7884 (113)	18.3
*Culex quinquefasciatus*	13 (1)	0	72 (1)	1.3	84 (1)	0.2
*Culex sp*	3	0	0	0	3	0
*Culex tigripes*	4	0	0	0	4	0
*Culex tritaeniorhynchus*	1470 (9)	3.9	176	3.1	1646 (9)	3.8
*Mansonia africana*	810 (162)	2.2	32	0.6	842 (162)	2
*Mansonia uniformis*	4110 (191)	11	112	2	4222 (191)	9.8
*Mimomyia mimomyiaformis*	57	0.2	0	0	57	0.1
*Mimomyia plumosa*	115 (1)	0.3	4	0.1	119 (1)	0.3
*Mimomyia splendens*	224 (34)	0.6	0	0	224 (34)	0.5
*Uranotaenia mayeri*	3	0	0	0	3	0
Total collected (males)	37392 (1070)	100	5600 (1)	100	42992 (1071)	100

**Fig. 2:**
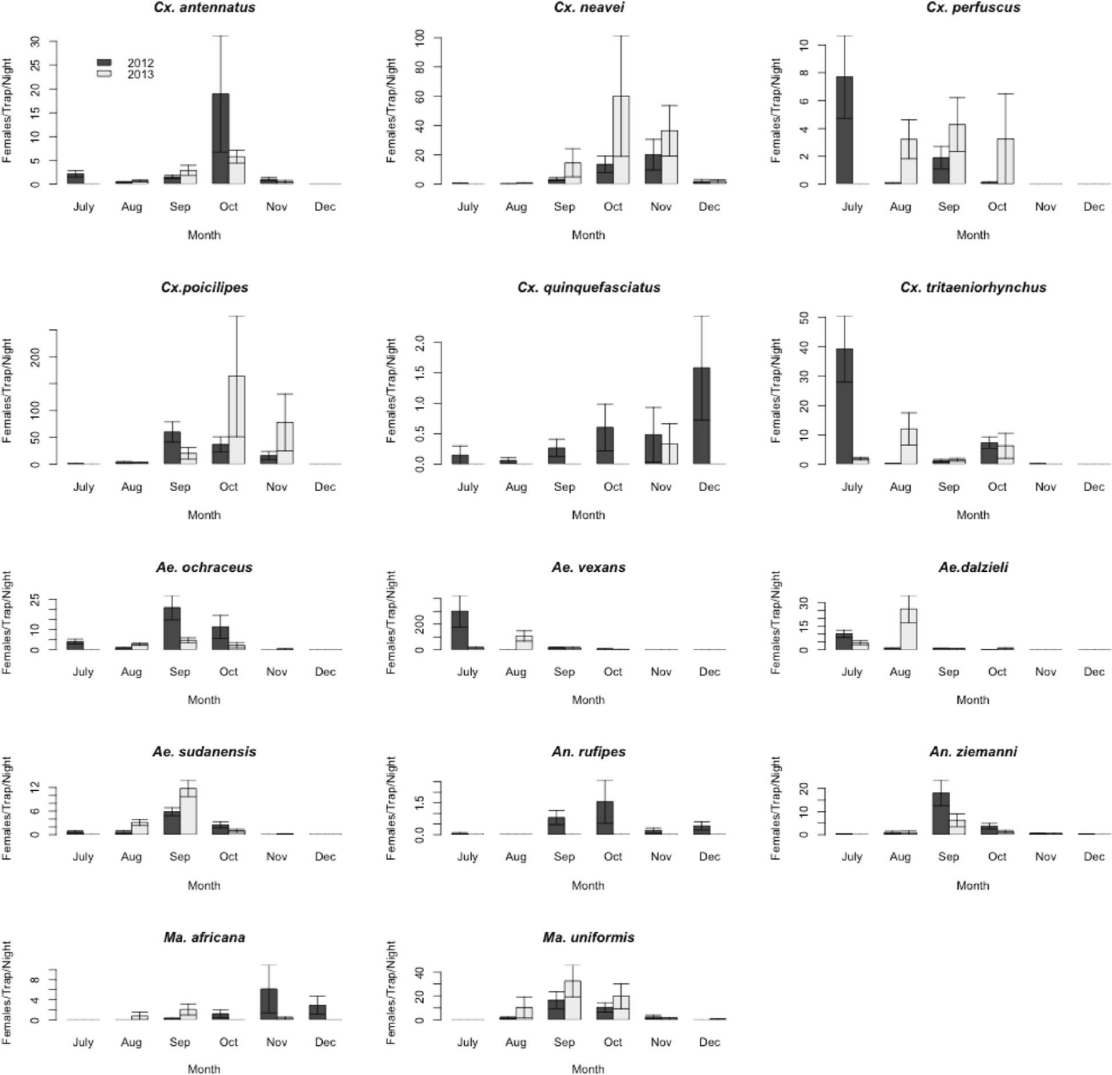
Temporal dynamics of mosquito vectors of arboviruses in the Barkedji area, 2012-2013

Only mosquito species (Table 2) found infected were considered in the following analyses. Proportions of each species varied significantly between ponds and villages (*p* < 0.0001). Seasonal patterns of mosquito females collected by CDC+CO_2_ light-traps are presented in Fig. 2. The largest numbers of females per CDC+CO_2_ trap per night were collected in October of both years for *Cx. antennatus*, November 2012 and October 2013 for *Cx. neavei*, September 2012 and October 2013 for *Cx. poicilipes*, July 2012 and September 2013 for *Cx. perfuscus*, November 2012 and September 2013 for *Ma. africana*, July 2012 and August 2013 for *Cx. tritaeniorhynchus*, July 2012 and August 2013 for *Ae. vexans* and *Ae. dalzieli*, September 2012 and 2013 for *Ae. ochraceus*, *Ae. sudanensis*, *An. ziemanni* and *Ma. uniformis,* October 2012 for *An. rufipes*.

**Table 2:** Viruses isolated, associated mosquitoes and vertebrates, and pathogen potential, Barkedji, 2012-2013

Virus	Year of isolation	Family	Mosquitoes found infected in this study	Vertebrates associated	Pathogen potential
Acado	2013	*Reoviridae*	*Culex antennatus, Cx. neavei, Mansonia uniformis*	Humans, domestic ungulates,	unknown
Bagaza	2012	*Flaviviridae*	*Culex antennatus, Cx. neavei*	Humans, wild birds, red-legged partridges and ring-necked pheasants, common wood pigeons	Unknown
	2013		*Cx. neavei*		
Barkedji	2012	*Flaviviridae*	*Cx. neavei, Ae. sudanensis*	Unknown	Unknown
	2013		*Cx. neavei*		
Ndumu	2012	*Togaviridae*	*Aedes dalzieli, Ae. vexans, Anopheles rufipes, An. ziemanni, Cx. poicilipes, Ma. uniformis*	Domestic pigs, Humans	Unknown
Rift valley fever	2013	*Bunyaviridae*	*Ae. ochraceus*		Yes
Sanar	2013	*Reoviridae*	*Cx. neavei, Ma. uniformis*	Unknown	Unknown
Sindbis	2012	*Togaviridae*	*Cx. antennatus, Cx. neavei*	Humans, Birds, Orangutans, Sheeps, frogs, reed warblers, Bats	Yes
	2013		*Cx. neavei*		
Usutu	2012	*Flaviviridae*	*Culex antennatus, Cx. neavei*	Human, African furred rat, blackbirds, Bats	Yes
	2013		*Cx. neavei*		
West Nile	2012	*Flaviviridae*	*Ae. dalzieli, An. rufipes, Cx. antennaus, Cx. neavei, Cx. perfuscus, Cx. poicilipes, Cx. quinquefasciatus*	Horses, Birds, Human	Yes
	2013		*Cx. neavei, Cx. tritaeniorhynchus*		
Yaounde	2012	*Flaviviridae*	*Cx. tritaeniorhynchus*	Bird (*Bycanistes sharpii*), Rodents (*Praomys sp*., *Cavia porcellus*)	Unknown

### Viruses

All the 42,969 mosquitoes in 4,429 pools were tested for virus isolation yielding 103 virus isolates within 10 virus species (Tables 2 and 3). WNV (30.1%), BKJV (17.5%), SINV (12.6%), and USUV (11.6%) were the most prevalent. All virus isolates were from 9 ponds (88.3 % of the virus and 50% of total ponds) and 7 villages (out of 31.8% of total villages sampled). Most of the isolates from ponds were from Kangaledji (59.3%), Fourdou (14,3%) and Niakha (13.2%). Three virus species (WNV, NDUV and SINV) were isolated from villages and 10 from ponds during the study period. The largest numbers of viruses were isolated in October (29.1% of total isolates) and November (50.5%). Viruses were isolated from 14 mosquito species. The largest numbers of viral isolations were from *Cx. neavei* (69.9% of the strains), *Cx. antennatus* (9.7%), and *Ma. uniformis* (4.8%). Seven different viral species were isolated from *Cx. neavei* (ACAV, BAGV, BKJV, SANV, SINV, USUV, and WNV), and *Cx. antennatus* (ACAV, BAGV, NDUV, SANV, SINV, USUV, and WNV) and 3 from *Ma. uniformis* (ACAV, NDUV and SANV). Viruses were detected from vectors before, during and after their peak abundances.

**Table 3:** Viruses isolated and minimum field infection rates by species, Barkedji area, 2012-2013

Species	ACAV		BAGV		BARV		NDUV		RVFV		SANV		SINV		USUV		WNV		YAOV		Total
	P+	MFIR	P+	MFIR	P+	MFIR	P+	MFIR	P+	MFIR	P+	MFIR	P+	MFIR	P+	MFIR	P+	MFIR	P+	MFIR	P+
*Aedes dalzieli*							1	0.7									1	0.7			2
*Aedes ochraceus*									1	0.5											1
*Aedes sudanensis*					1	1.4															1
*Aedes vexans*							1	0.9													1
*Anopheles rufipes*							1	10.0									1	10.0			2
*Anopheles ziemanni*							1	0.4													1
*Culex antennatus*	1	0.7	1	0.7			1	0.7			1	0.7	1	0.7	2	1.5	3	2.2			10
*Culex neavei*	5	1.3	2	0.5	16	4.3					4	1.1	12	3.2	9	2.4	22	5.8			70
*Culex perfuscus*																	1	1.8			1
*Culex poicilipes*							1	0.1									1	0.1			2
*Culex quinquefasciatus*																	1	12.5			1
*Culex tritaeniorhynchus*																	1	0.6	1	0.6	2
*Mansonia africana*	1	1.5									1	1.5									2
*Mansonia uniformis*							3	0.7													3
*Culex neavei* male					1										1						2
*Mansonia uniformis* male	1										1										2
Total	8		3		18		9		1		7		13		12		31		1		103

P+: number of positive pools; MFIR: minimum field infection rate (total number positive pools / the total number of mosquitoes tested *1000)

### West Nile virus

**Fig. 3:**
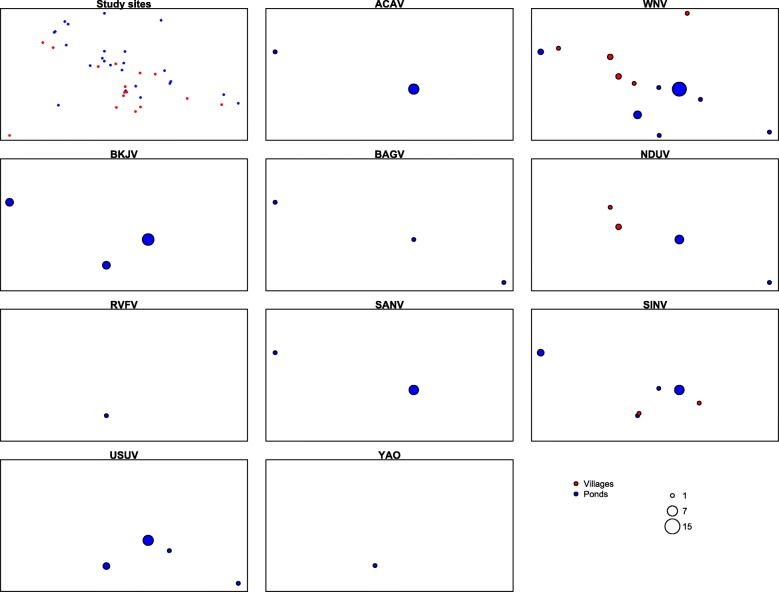
Spatial distribution of arboviruses detected in ponds and villages of the Barkedji area, 2012-2013. Saaka pond, from which Barkedji and West Nile viruses were isolated (1 pool each) is not included in the map because it is located about 50Km from the other sites

Overall, WNV was isolated from 31 mosquito pools belonging to 3 genera and 8 species collected in 8 ponds (24/77.4% of all the WNV strains) and 5 villages (Table 4 & Fig. 3). The largest numbers of WNV isolates were from the Kangaledji ponds (54.2% of the strains detected in ponds). The virus was identified from 22 pools of *Cx. neavei* (71%), 3 pools of *Cx. antennatus* (9.7%) and 1 pool of each of the 6 other vectors (*Cx. poicilipes*, *Cx. perfuscus*, *Cx. tritaeniorhunchus*, *Cx. quinquefasciatus*, *Ae. dalzieli* and *An. rufipes*). Infected pools were collected in July (2 pools / 6.4 % of the total) September (3 pools / 9.7 %), October (12 pools / 38.7 % and November (14 pools / 45.2 %). The WNV MFIR per thousand pooled mosquitoes for all species are presented Tables 3 and 4. Mean minimum field infection rates among species (Table 3), differed significantly (χ^2^ = 54.8, DF = 7, *p* < 0.0001) among mosquito vectors, ranging from 12.5 ‰ for *Cx. quinquefasciatus* to 0.1 ‰ for *Cx. poicilipes*. The highest infection rate was observed in November for *Cx. neavei* and *Cx. antennatus* (Table 4). Differences across the season in MFIR were not statistically significant for *Cx. neavei* and *Cx. antennatus* (*p* > 0.2).

**Table 4:** Viruses isolated in mosquito females and minimum field infection rates by month, Barkedji area, 2012-2013

Virus		July				September				October				November				December			
	Species	No	NP	P+	MFIR	No	NP	P+	MFIR	No	NP	P+	MFIR	No	NP	P+	MFIR	No	NP	P+	MFIR
WNV	*Aedes dalzieli*	367	52	0	0.0	108	46	0	0.0	7	6	1	142.9	0	0	0	0.0	0	0	0	0.0
	*Anopheles rufipes*	1	1	0	0.0	33	9	0	0.0	61	10	1	16.4	8	6	0	0.0	8	6	0	0.0
	*Culex antennatus*	46	15	0	0.0	176	77	0	0.0	990	71	2	2.0	96	35	1	10.4	0	0	0	0.0
	*Culex neavei*	12	6	0	0.0	669	97	3	4.5	1193	78	6	5.0	1721	128	13	7.6	104	25	0	0.0
	*Culex perfuscus*	155	16	1	6.5	222	64	0	0.0	64	21	0	0.0	0	0	0	0.0	0	0	0	0.0
	*Culex poicilipes*	19	6	0	0.0	3168	149	0	0.0	2401	114	1	0.4	1974	113	0	0.0	4	4	0	0.0
	*Culex quinquefasciatus*	4	2	1	250.0	13	6	0	0.0	23	3	0	0.0	20	5	0	0.0	20	5	0	0.0
	*Culex tritaeniorhynchus*	842	48	0	0.0	98	40	0	0.0	343	47	1	2.9	14	12	0	0.0	0	0	0	0.0
	Total vectors	1446	146	2	1.4	4487	488	3	0.7	5082	350	12	2.4	3833	299	14	3.7	136	40	0	0.0
USUV	*Culex antennatus*	46	15	0	0.0	176	77	0	0.0	990	71	1	1.0	96	35	1	10.4	0	0	0	0.0
	*Culex neavei*	12	6	0	0.0	669	97	3	4.5	1193	78	2	1.7	1721	128	4	2.3	104	25	0	0.0
	Total vectors	58	21	0	0.0	845	174	3	3.6	2183	149	3	1.4	1817	163	5	2.8	104	25	0	0.0
ACAV	*Culex antennatus*	46	15	0	0.0	176	77	0	0.0	990	71	0	0.0	96	35	1	10.4	0	0	0	0.0
	*Culex neavei*	12	6	0	0.0	669	97	0	0.0	1193	78	1	0.8	1721	128	4	2.3	104	25	0	0.0
	*Mansonia africana*	0	0	0	0.0	64	30	0	0.0	76	17	0	0.0	256	31	0	0.0	263	20	1	3.8
	Total vectors	58	21	0	0.0	909	204	0	0.0	2259	166	1	0.5	2073	194	5	2.8	367	45	1	2.7
BAGV	*Culex antennatus*	46	15	0	0.0	176	77	0	0.0	990	71	1	1.0	96	35	0	0.0	0	0	0	0.0
	*Culex neavei*	12	6	0	0.0	669	97	0	0.0	1193	78	0	0.0	1721	128	2	1.2	104	25	0	0.0
	Total vectors	58	21	0	0.0	845		0	0.0	2183	149	1	0.5	1817	163	2	1.1	104	25	0	0.0
BARV	*Aedes sudanensis*	15	8	0	0.0	517	103	1	1.9	111	36	0	0.0	2	2	0	0.0	0	0	0	0.0
	*Culex neavei*	12	6	0	0.0	669	97	2	3.0	1193	78	4	3.4	1721	128	10	5.8	104	25	0	0.0
	Total vectors	27	14	0	0.0	1186	200	3	2.5	1304	114	4	0.0	1723	130	10	0.0	104	25	0	0.0
NDUV	*Aedes dalzieli*	367	52	0	0.0	108	46	0	0.0	7	6	1	142.9	0	0	0	0.0	0	0	0	0.0
	*Aedes vexans*	6603	187	0	0.0	1408	102	0	0.0	278	44	1	3.6	4	4	0	0.0	0	0	0	0.0
	*Anopheles rufipes*	1	1	0	0.0	33	9	0	0.0	61	10	1	16.4	8	6	0	0.0	8	6	0	0.0
	*Anopheles ziemanni*	6	6	0	0.0	1879	129	1	0.5	434	44	0	0.0	62	33	0	0.0	10	8	0	0.0
	*Culex antennatus*	46	15	0	0.0	176	77	0	0.0	990	71	0	0.0	96	35	0	0.0	0	0	0	0.0
	*Culex poicilipes*	19	6	0	0.0	3168	149	0	0.0	2401	114	1	0.4	1974	113	0	0.0	4	4	0	0.0
	*Mansonia uniformis*	0	0	0	0.0	2277	142	3	1.3	864	70	0	0.0	127	30	0	0.0	2	2	0	0.0
	Total vectors	7042	267	0	0.0	9049	654	4	0.4	5035	359	4	1.0	2271	221	0	0.0	24	20	0	0.0
SANV	*Culex antennatus*	46	15	0	0.0	176	77	0	0.0	990	71	0	0.0	96	35	1	10.4	0	0	0	0.0
	*Culex neavei*	12	6	0	0.0	669	97	0	0.0	1193	78	0	0.0	1721	128	4	2.3	104	25	0	0.0
	*Mansonia africana*	0	0	0	0.0	64	30	0	0.0	76	17	0	0.0	256	31	0	0.0	263	20	1	3.8
	Total vectors	58	21	0	0.0	909	204	0	0.0	2259	166	0	0.0	2073	194	5	2.8	367	45	1	2.7
SINV	*Culex antennatus*	46	15	0	0.0	176	77	0	0.0	990	71	0	0.0	96	35	1	10.4	0	0	0	0.0
	*Culex neavei*	12	6	0	0.0	669	97	1	1.5	1193	78	5	4.2	1721	128	6	3.5	104	25	0	0.0
	Total vectors	58	21	0	0.0	845	174	1	1.2	2183	149	5	2.3	1817	163	7	3.9	104	25	0	0.0
RVFV	*Aedes ochraceus*	83	17	0	0.0	1250	110	1	0.8	516	53	0	0.0	5	4	0	0.0	0	0	0	0.0
YAOV	*Culex tritaeniorhynchus*	842	48	1	1.2	98	40	0	0.0	343	47	0	0.0	14	12	0	0.0	0	0	0	0.0

No: Number of female mosquitoes collected; NP: Number of pools; P+: number of positive pools; MFIR: minimum field infection rate (total number positive pools / the total number of mosquitoes tested *1000)

### Usutu virus

>This virus was isolated from 10 pools (9 females and 1 male) of *Cx. neavei* (83.3% of the isolates) and 2 pools of *Cx. antennatus* collected in 4 ponds (Table 4 & Fig. 3). The largest numbers of viral isolates were from the Kangaledji pond with 7 pools (58.3% of the strains; Fig. 3). Infected pools were collected in September (3 pools), October (3) and November (5) (Table 4). The mean minimum infection rates of *Cx. antennatus* (1.5‰) and *Cx. neavei* (2.4‰) (Table 3) were comparable (*p* = 0.7). The highest infection rate was observed in November for *Cx. antennatus* and September for *Cx. neavei*. The differences in variation in MFIR across the season were not statistically significant for *Cx. neavei* and *Cx. antennatus* (*p* > 0.2).

### Acado virus

This virus was isolated from 5 pools of *Cx. neavei* (71.4% of the isolates), 1 pool of both *Cx. antennatus* and *Ma. africa* and 1 pool of *Ma. uniformis* male (Table 3) collected from the Kangaledji and Niakha ponds (Fig. 3). Infected pools were collected in October (1 pool), November (5 pools) and December (1 pool) (Table 4). The mean MFIR of *Cx. antennatus* (0.7 ‰) and *Cx. neavei* (1.3 ‰) presented in Table 3 were comparable (*p* = 1).

The highest infection rate was observed in late November for *Cx. neavei*. The differences were not statistically significant (*p* = 0.86). The differences of the seasonal variation in MFIR were not statistically significant for *Cx. neavei* (*p* > 0.09)

### Bagaza virus

This virus was isolated from 2 pools of *Cx. neavei* and 1 pool of *Cx. antennatus* (Tables 2 and 3) collected in 3 ponds (Fig. 3). Infected pools were collected in October (1 pool) and November (2 pools) (Table 4). The mean MFIR of *Cx. antennatus* (0.7 ‰) and *Cx. neavei* (0.5 ‰) were comparable (*p* = 1).

### Barkedji virus

This virus was isolated from *Cx. neavei* (17 pools/16 females and 1 male), and *Ae. sudanensis* (1 pool) collected in 4 ponds (Table 3 & Fig. 3). The largest numbers of viral strains were from the Kangaledji pond with 9 pools (52.9 % of the strains). Positive pools were found in September (3 pools) October (4 pools), and November (10 pools) (Table 4). The highest seasonal infection rate was observed in November but the difference with the other months was not statistically significant (*p* = 0.6).

### Ndumu virus

A total of 9 NDUV strains were isolated from mosquito pools belonging to 4 genera and 7 species (Tables 2 and 3) collected in 2 ponds (6/66.7% of all strains) and 2 villages (Fig. 3). The largest numbers of viral strains were from the Kangaledji pond (83.3% of the strains collected in ponds). The virus was identified from *Ma. uniformis* (3 pools/33.3% of the total), *Ae. dalzieli*, *Ae. vexans*, *An. rufipes*, *An. ziemanni Cx. antennatus* and *Cx. poicilipes* (1 pool each). Infected pools were collected in September (5 pools) and October (4 pools). No species showed seasonal variation of it MFIR (Table 4).

### Sanar virus

This virus was isolated from *Cx. neavei* (4 pools; 57.1 % of the strains), *Cx. antennatus, Ma. africana* and *Ma. uniformis* male (1 pool for each species) collected from the Kangaledji (6 pools) and Niakha (1pools) pond (Table 3 & Fig. 3). Infected female pools were collected in November (5 pools) and December (1 pool) (Table 4). The mean MFIR of *Cx. antennatus* (0.7‰) and *Cx. neavei* (1.1‰) were comparable (*p* = 1).

### Sindbis virus

SINV was isolated from 12 pools of *Cx. neavei* and 1 pool of *Cx. antennatus* (Tables 2 and 3) collected in 4 different ponds and 2 villages (Fig. 3). Infected pools were collected in September (1 pool), October (5 pools) and November (7 pools). The mean MFIR of *Cx. antennatus* (0.7‰) and *Cx. neavei* (3.2‰) were comparable (*p* = 0.2).

### Yaounde virus

Only one isolate of this virus was recovered from a pool of *Cx. tritaeniorhynchus* (Tables 2 and 3) collected in a pond on July (Table 4 & Fig. 3).

### Rift valley fever virus

The single strain of RVFV was isolated from a pool of *Ae. ochraceus* collected from a pond in September 2013 (Table 4 & Fig. 3).

## Discussion

In this study we detected 10 arbovirus species from the Barkedji area, emphasizing the importance of this area as a hotspot of enzootic arboviruses transmission and a good platform for vector-borne diseases surveillance [1]. Moreover, we detected NDUV, ACAV, and SINV for the first time in the Barkedji area after more than 20 years of arbovirus surveillance, demonstrating the high biodiversity of arboviruses in the region and suggesting that our knowledge of the arboviruses present is still probably incomplete. Our study also highlights the importance of using diverse sampling methods and sites to characterize vector and arbovirus diversity. WNV, NDUV and SINV were the only three virus species isolated from villages, suggesting that these viruses pose a particular risk for transmission to human and domestic animals in the Barkedji area.

ACAV and SANV were isolated from a pool of male *Ma. uniformis* and USUV and BARV from a pool of male *Cx. neavei*, representing the first time in our knowledge, that these viruses have been detected in male mosquitoes, supporting the possibility of the maintenance of these viruses by vertical transmission in the wild. Thus, these mosquito species may be investigated as reservoirs of these viruses during the dry season.

RVFV has been isolated from several mosquito species in many African countries [1, 29]. This virus has been responsible for widespread outbreaks in both humans and domestic ungulates in Africa and the Arabian Peninsula [30–36]. The disease is characterized by mass abortions and high mortality in animals resulting in high economic losses [4]. In humans also, high mortality rates and severe complications have been observed [37]. Detection of a single isolate of RVFV in September 2013 in our study, was followed by several outbreaks of RVFV in human and ungulates in others parts of Senegal (http://www.oie.int/wahis_2/public/wahid.php/Reviewreport/Review?reportid=14211). This very low amplification of RVF did not involve *Ae. vexans* and *Cx. poicilipes*, known as the most abundant species and the main vectors of RVFV in West Africa [13, 18]. This single isolation of RVFV may also suggest that the amplification was too low to spill over to human and domestic ungulates in the Barkedji area.

This study provides the first documented evidence of ACAV in Senegal. ACAV, like SANV, is a member of the *Corriparta virus* species/serogroup (family: *Reoviridae*, genus: *Orbivirus*). This virus was first isolated in 1963 from a mixed *Culex antennatus* and *Cx. neavei* pool collected in the Acado Baro River, Ilubabor Pro in Ethiopia [38]. ACAV has never been associated to any vertebrates but mosquito species infected by ACAV are ornithophagic and have been associated with pathogenic viruses. Thus, ACAV may be potentially pathogenic to human and animals in Barkedji. This virus was detected in only 2 of the ponds investigated and mainly in November indicating a highly focal spatio-temporal amplification pattern.

SANV was isolated for the first time in 1990 from *Cx. poicilipes* in Dakar-Bango near Saint Louis, Senegal, and later several other mosquitoes in the Senegal River Basin, Kedougou, and Barkedji area [1, 18]. In this study, the virus was isolated mainly from *Cx. neavei*, which should be considered as the main vector. *Cx. antennatus*, *Ma. africana*, and *Ma. uniformis* are potential secondary vectors. Its vertebrate hosts and disease association are unknown. However, the ornitophagic tendency of the SANV vectors suggest that birds may play a potential role in the natural history of this virus. Viruses belonging to *Corriparta virus* serogroup detected in Australia, Africa and South America have been detected in a wide range of vertebrate hosts [39–42].

Five members of the genus *Flavivirus*, family *Flaviviridae* (WNV, USU, BAGV, BARV, and YAOV) were isolated in our study. WNV was isolated from 31 mosquito pools (belonging to 8 species), 8 ponds and 5 villages confirming the high and wide enzootic activity of the virus in the area [1, 43]. Indeed, this virus was continuously isolated from mosquitoes collected in the area and antibodies directed against the virus were detected in more than 78% of horses, predicted in up to 39% of resident birds from Barkedji [44, 45]. Antibodies directed against WNV were detected in 80% of a human sample collected in a village located at 80 km from Barkedji [1]. Human disease attributable to WNV in Africa has most often been categorized as mild febrile illness with rash, although more severe symptoms including hepatitis and encephalitis have been reported [46, 47]. WNV was isolated mainly from *Cx. neavei* and from *Cx. antennatus*, *Cx. poicilipes*, *Cx. perfuscus*, *Cx. tritaeniorhunchus*, *Cx. quinquefasciatus*, *Ae. dalzieli* and *An. rufipes*. WNV was isolated previously from *Cx. neavei*, *Cx. perfuscus* and *Cx. tritaeniorhynchus* in Barkedji and from *Cx. antennatus* and *Ma. uniformis* in the Senegal River Basin [18, 43] and *Ae. dalzieli* in Kedougou [43]. The virus was detected for the first time from *Cx. antennatus*, *Ae. dalzieli* and *Ma. uniformis* in Barkedji and from *Cx. quinquefasciatus* in Senegal. Vector competence of *Cx. neavei, Cx. quinquefasciatus, Cx. tritaeniorhunchus* and *Ae. vexans* for WNV has been demonstrated [48–50]. Moreover, *Cx. neavei* is mainly ornithophagic but feed also to a lesser extent on humans, cattle and horses [51–53], and *Cx. antennatus* is zoophagic but feeds occasionally on humans, cattle and birds [17, 54]. These facts suggest that *Cx. neavei* should be considered as the principal enzootic vector and *Cx. antennatus* an epizootic vector of WNV in Barkedji. Contrary to our results, most of the WNV strains isolated in previous studies were from *Cx. poicilipes* in the Barkedji area [1, 43] and from *Ma. uniformis* in the Senegal River Basin [18], suggesting that the importance a given species as a vector could vary in space and time.

USUV was detected mainly from *Cx. neavei* in temporary ponds in concordance with previous isolation of USUV in Senegal and South Africa, and the enzootic and sylvatic pattern of its transmission in Africa [55]. USUV been isolated from an African furred rat, and a human with fever and rash as clinical symptoms. Considering its bionomics and the virus isolations, we should consider *Cx. neavei* as the main enzootic and bridge vector of USUV in Senegal.

BAGV was detected from *Cx. neavei* and *Cx. antennatus* in ponds in this study, suggesting that the virus circulates in an enzootic cycle between these ornithophagic mosquitoes and birds within ponds. This virus has a wide distribution and was recovered from several West African countries, South Africa, Spain, Israel and India [55–57]. It was first isolated in 1966 in the Bagaza province of the Central African Republic from *Culex* spp. and later from several mosquito species in Senegal and other West African countries [55, 58]. This virus has also been isolated from *Cx. tritaeniorhynchus* from India [57]. Vector competence of *Cx. tritaeniorhynchus*, *Cx. quinquefasciatus* and *Ae. aegypti* and the vertical transmission of the virus by *Cx. tritaeniorhynchus* have been demonstrated in India [56]. In the current study, *Cx. neavei* and *Cx. antennatus* could be considered as probable vectors of BAGV. BAGV was responsible for febrile illness in humans and high mortality to wild birds in Spain [59]. Antibodies to the virus have been also found in 15 % of human samples from India [57].

Most of BARV strains were isolated in *Cx. neavei.* This virus was isolated for the first time in Barkedji and then later isolated in *Culex perexiguus* in Israel in 2011 [59]. BARV virus has never been isolated from human or other vertebrate hosts. However, the ornithophagic tendency [17, 51, 53] of its vectors suggest that birds may play a potential role in the natural history of this virus.

YAOV was first isolated in 1968 from *Cx. nebulosus* in Cameroon [27]. Later, the virus was isolated from several other mosquito species in Senegal and other West African countries [60]. YAOV was isolated from *Cx. tritaeniorhynchus* for the first time, in our knowledge, in our study. YAOV has been isolated from one bird (*Bycanistes sharpii*) two rodents (*Praomys sp*. and *Cavia porcellus*) but this virus has never been detected in humans.

NDUV and SINV are the two members of the genus *Alphavirus*, family: *Togaviridae* identified in this study. NDUV was identified from mainly *Ma. uniformis* suggesting that it was the main vector of this virus. NDUV was first isolated in 1959 from *Ma. uniformis* in South Africa and later from mosquitoes and ticks in other African countries [11, 12]. NDUV was also isolated from 2 pools of *Cx. pipiens* females collected as larvae in Kenya, suggesting for the first time a natural vertical transmission of this virus [61]. This virus was isolated from villages in our study, suggesting the possible involvement of domestic animals and/or humans in the transmission cycle. This assertion is supported by detection of NDUV in domestic pigs in Uganda and antibodies to the virus in humans from several African countries [62–64].

SINV was originally isolated from *Cx. pipiens* and *Cx. univittatus* collected in a village named Sindbis in Egypt in 1952 [65]. Later, the virus was detected from several mosquito species in Africa, and several mosquito and tick species outside of Africa [66]. Vector competence of *Cx. neavei* for SINV has been demonstrated in South Africa [49]. In this study, we reported SINV mainly in *Cx. neavei* and a single pool of *Cx. antennatus* for the first time in the Barkedji Area. Infected mosquitoes have been found in ponds and villages supporting the possible involvement of birds, domestic animals and humans in the transmission cycle of this virus. Indeed, SINV seropositivity in humans has been reported in various areas of Africa, Australia, Northern Europe, and the Middle East, and antibodies to SINV have also been found from various vertebrates including birds [67] orangutans [68] and sheep [69]. The virus has been isolated from frogs [70], reed warblers [71], bats [72], and humans [67, 73]. SINV symptoms of infection in humans include fever, arthritis, rash, tenderness and persistent arthralgia [74].

## Conclusion

This paper described an update of a mosquito-based arbovirus surveillance system, with more sampling methods and sites included, in the Barkedji area. Ten virus species, including 3 new for the study area, were detected, indicating a high biodiversity of viruses of medical, veterinary and unknown importance in this area. The distribution of arboviruses, detected in 14 mosquito species, was highly focal in space and time, and was highest in October and November. WNV outbreaks in the Americas have shown that arboviruses considered of little importance can emerge quickly and invade globally. Because the Barkedji area hosts a high number of arboviruses with potential to emerge, this area, and others like it, should be consistently surveilled in order to be well-prepared to deal with future invaders.

## Abbreviations

ACAV: Acado virus
Ae: Aedes
An: Anopheles
AP61: Aedes pseudoscutellaris
BAGV: Bagaza virus
BARV: Barkedji virus
Cx: Culex
Er: Eretmapodites
Ma: Mansonia
MFIR: Minimum field Infection Rate
Mi: Mimomyia
NDUV: Ndumu virus
RVFV: Rift valley fever virus
SANV: Sanar virus
SINV: Sindbis virus
USUV: Usutu virus
WHO: World Health Organization
WNV: West Nile virus
YAOV: Yaounde virus
